# A Comparison of asIgE Levels Measured with ALEX and ImmunoCAP ISAC in Polish Children with Food Allergies

**DOI:** 10.3390/ijms26051810

**Published:** 2025-02-20

**Authors:** Emilia Majsiak, Magdalena Choina, Karolina Miśkiewicz, Solomiya Pukalyak, Sylwia Smolińska, Ryszard Kurzawa

**Affiliations:** 1 Department of Health Promotion, Faculty Health of Sciences, Medical University of Lublin, Staszica 4/6, 20-081 Lublin, Poland; 2 Department of Experimental Allergology and Immunology, Medical University of Bialystok, 15-090 Bialystok, Poland; ma.choina@o2.pl; 3 Department of Allergology and Internal Medicine, Medical University of Bialystok, M. Skłodowskiej-Curie 24A, 15-276 Białystok, Poland; 4 Department of Allergy and Pulmonology, Institute of Tuberculosis and Lung Diseases Regional Branch in Rabka-Zdrój, prof. Jana Rudnika 3B, 34-700 Rabka-Zdrój, Poland; kmiskiewicz@igrabka.edu.pl (K.M.); ryszard.kurzawa@gmail.com (R.K.); 5 The Polish–Ukrainian Foundation of Medicine Development, Nałęczowska 14, 20-701 Lublin, Poland; solomiya.pukalyak@gmail.com; 6 Department of Clinical Immunology, Faculty of Medicine, Wroclaw Medical University, Parkowa 34, 51-616 Wroclaw, Poland

**Keywords:** ALEX, ImmunoCAP ISAC, allergen-specific immunoglobulin E (asIgE), molecular allergy diagnostics

## Abstract

In order to establish the reliability of new multiplex tests for determining allergen-specific immunoglobulin E (asIgE) levels in serum, the results of asIgE determinations obtained via ALEX tests were compared to those obtained via ImmunoCAP ISAC in a group of 40 Polish children hospitalized due to food allergy and/or anaphylaxis. The analysis was based on 6320 determinations of asIgEs relative to 79 common allergen molecules included in both tests (80 tests conducted—40 ALEX and 40 ISAC). The study showed a high correlation of results between the compared diagnostic tests, with the concordance of asIgE determinations at the level of 92.5% (*n* = 2922). The results of this study showed the sensitivity of the ALEX test to be comparable with that of the ImmunoCAP ISAC test. One indisputable advantage of the ALEX test is its high number of determinations per procedure, which provides more information about the patient’s molecular profile; moreover, the use of an inhibitor of the asIgEs that bind to cross-reactive carbohydrate determinants (CCDs) reduces their impact on the results for allergens naturally containing carbohydrate determinants. Comparative analyses of asIgE test results obtained with ALEX and ImmunoCAP ISAC tests demonstrate that the ALEX test is a reliable diagnostic tool for asIgE assessments. However, significant differences in the absolute values of asIgEs relative to individual molecules suggest the need for caution when directly comparing asIgE levels from both tests.

## 1. Introduction

Molecular diagnostics has opened a new chapter in allergy management by facilitating a more personalized approach [1]. Multiplex allergen-specific immunoglobulin E (asIgE) testing helps fully utilize the potential of personalized medicine [2]. This novel diagnostic tool allows us to detect the similarities and differences in the allergy profiles of various populations [3]. Large-scope studies with the use of multiplex testing may help determine which allergen extracts and molecules are of clinical significance in a given region.

The first tool, the ImmunoCAP Immuno Solid-Phase Allergen Chip (ISAC) test, became available for commercial use in 2011. This test offers a semi-quantitative assessment of serum asIgEs relative to 112 (67 recombinant and 45 natural) allergen molecules derived from 51 sources [4,5,6]. The next multiplex test, the Allergy Xplorer (ALEX) test, was presented for the first time in 2017 at the annual congress of the European Academy of Allergy and Clinical Immunology (EAACI) held in Helsinki. The multiplex third-generation ALEX test is an immune-enzyme assay, which can determine asIgEs relative to 282 allergen components derived from 167 sources. A single assay determines asIgEs relative to 156 allergen extracts and 126 molecules (80 recombinant molecules, 44 natural molecules, and 2 mixed, part recombinant–part natural protein) simultaneously. ALEX testing makes use of nanoparticle-bound allergen extracts and molecules placed in a solid phase, which forms a macroscopic matrix, to which a cross-reactive carbohydrate determinant (CCD) inhibitor and a sample of the patient’s serum are added [7]. The use of nanoparticles for testing and a sensitive asIgE detection system helps lower the required allergen quantity considerably without compromising test accuracy [8]. The use of nanoparticles improves test sensitivity since it ensures the three-dimensional presentation of a higher number of epitopes of a given allergen than would be possible by placing them on a flat membrane of equal area [8]. This is particularly important in the case of low asIgE levels and has made it possible to include approximately 300 evaluable parameters in a single assay [9].

Due to the current significance of the molecular diagnostics of allergies, research studies comparing the usefulness of various diagnostic tests play an important role in identifying the diagnostic tools that would be most clinically applicable in specific populations. Moreover, the results of such studies may be a basis for developing new guidelines for allergy management, particularly with respect to diagnostic and therapeutic aspects [8].

Given the relatively recent introduction of the ALEX test, there is still a scarcity of studies comparing it with other multiplex tests [9]. Importantly, in order to assess and use molecular allergy profiles obtained via the ALEX test, it is necessary to verify the reliability of this test in any given population. To our knowledge, in Poland, there have been no recent studies comparing the results obtained via the ALEX test with those obtained via other diagnostic tools. Therefore, the aim of this study was to compare the asIgE values determined via the ALEX test with those obtained via the ImmunoCAP ISAC test in Polish children hospitalized due to food allergy.

All researchers who attempted to compare asIgE determinations obtained via the ALEX test with those obtained via other multiplex tests achieved promising results [3,10,11,12,13]. Nonetheless, such data are not available for Poland. To our knowledge, our study is one of the first ones to compare asIgE assessments obtained via the ALEX test with those obtained via the ImmunoCAP ISAC test in a Polish pediatric population. This is also one of the first studies to compare the results involving such a large number of allergen molecules common to both tests. Such broad allergy profiles facilitate a better understanding of the nature of allergic conditions and, consequently, an introduction of therapeutic recommendations that are better suited to patients’ needs [1,2,3,10].

## 2. Results

We compared 3160 determinations conducted with the ALEX test and 3160 determinations conducted with the ImmunoCAP ISAC test that quantified asIgEs to the same 79 allergen molecules included in both tests (80 tests conducted—40 ALEX and 40 ISAC). The ALEX test showed 2515 negative results (79.6%) and 645 positive results (20.4%). The ImmunoCAP ISAC test showed 2493 negative results (78.9%) and 667 positive results (21.1%). The chi-square test of independence showed no statistically significant differences in the results (asIgEs present/asIgEs absent) obtained with the two tests (*p* = 0.495) (Table 1).

Analyses of the results obtained with ALEX and ImmunoCAP ISAC tests revealed 2922 (92.5%) concordant asIgE results, 537 of which (17.0%) were positive and 2385 (75.5%) were negative. Non-concordant results were observed for 238 asIgE determinations (7.5%). These included 108 positive ALEX test results that were negative in the ImmunoCAP ISAC test and 130 positive ImmunoCAP ISAC test results that were negative in the ALEX test (Table 2).

**Figure 1:**
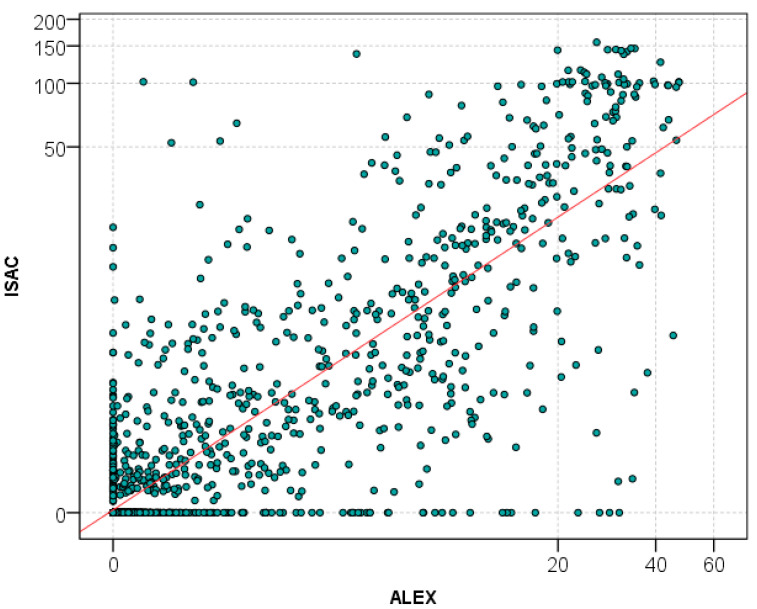
Point distribution of asIgE results obtained via ImmunoCAP ISAC and ALEX tests on a logarithmic scale.

Analysis of all obtained data showed a high Spearman’s rank correlation between ImmunoCAP ISAC and ALEX test results (*r* = 0.73; *p* < 0.001) (Figure 1). Moreover, there was a high correlation of concordant positive asIgE results (*n* = 537) (Spearman’s rank correlation coefficient *r* = 0.72; *p* < 0.001).

Out of the 79 assessed allergen molecules, the highest concordance of asIgE results (100%) was observed for 9 molecules (11%): Ani s 1, Ani s 3, Bla g 5, Can f 1, Der f 2, Der p 2, Fag e 2, Hev b 5, and Pla a 1. Fifty molecules (63%) showed a concordance between 90% and 99%; seventeen molecules (18%) showed a concordance between 80% and 89%. The lowest asIgE result’s concordance was observed in terms of molecules Ara h 1 (75%), Ara h 3 (75%), and Jug r 2 (70%) (Table 3). A detailed analysis of compliance for the 79 analyzed molecules in the ALEX and ISAC tests is presented in Table A1.

**Figure 2:**
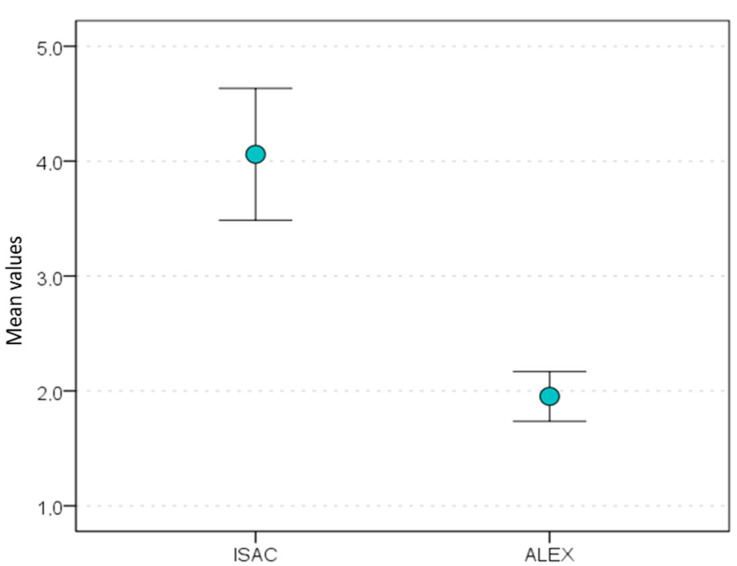
The mean test results with a 95% confidence interval.

The mean ALEX test result was 2.0 kU_A_/L (SD = 6.22), with the lowest and highest asIgE levels of 0.3 kU_A_/L and 47.1 kU_A_/L, respectively. The ImmunoCAP ISAC test yielded a mean asIgE level of 4.1 ISU (SD = 16.44) within a range from 0.3 ISU to 156.0 ISU. The Mann–Whitney U test showed the difference in mean asIgE levels between the evaluated tests to be statistically significant (*Z* = −7.88; *p* < 0.001) (Figure 2).

## 3. Discussion

Our analysis of asIgE determinations with the ALEX and ImmunoCAP ISAC tests showed an overall high concordance of over 92%. For 11% of the 79 evaluated molecules, the inter-test concordance was 100%, and concordance of over 90% was obtained for a total of 75% of the results. Study data analysis revealed a strong correlation between the ImmunoCAP ISAC and ALEX tests in terms of all asIgE determinations, both concordantly positive and concordantly negative, which indicates a high reliability of both tests in allergy assessment. The data analysis revealed a strong correlation between the ImmunoCAP ISAC and ALEX tests regarding all asIgE determinations, both concordantly positive and negative. Notably, only 7.5% of the results were discordant. Possible reasons for the discrepancies observed include differing specificity and sensitivity profiles between the two test and variations in the representation of epitopes within individual allergens or the type of molecules analyzed (whether recombinant or natural). Furthermore, the presence of blocker substances against carbohydrate residues, such as CCD (cross-reactive carbohydrate determinants), may influence the detection of IgE responses, leading to divergent results. Despite these potential factors contributing to the 7.5% discordance, the overall high concordance between both methods is underscored by the fact that the chi-square test of independence did not reveal any statistically significant differences between the results obtained from both methods (*p* = 0.495).

A comparison of the results revealed statistically significant differences in the distribution of asIgE levels obtained with the ALEX and ImmunoCAP ISAC tests, which suggests different limits of detection, despite an overall high concordance. Therefore, despite a strong correlation between ALEX and ImmunoCAP ISAC test results, the asIgE levels obtained with the two tools are not directly comparable. The observed discrepancies may be due to the differences in quantitative and semi-quantitative characteristics and the upper limit of quantification of either test. However, the obtained data suggest that ALEX test results correlate with ImmunoCAP ISAC test results, and the test itself is a reliable tool for asIgE assessment, which supports its usefulness in allergy diagnostics.

The results of the first study comparing asIgE determination via the ALEX and ImmucoCAP ISAC tests in Polish patients were presented at the 2019 EAACI congress in Lisbon [14]. The study population comprised 20 Polish children hospitalized due to food allergy and/or anaphylaxis at the Institute of Tuberculosis and Lung Disease Department of Allergy and Pulmonology in Rabka-Zdrój (the serum asIgE levels of those children have been also included in our present study). The reported concordance of asIgE quantification via the ALEX and ImmunoCAP ISAC tests in that group of 20 Polish patients was 91.3%. In the present study, which included a two times greater study population, the concordance of asIgE results was slightly higher. In the previous report (in Lisbon), the asIgE determinations characterized by the lowest concordance were observed for Ara h 1, Cor a 8, and Ara h 3 molecules (75%, 75%, and 55%, respectively), whereas in the present study, the lowest asIgE concordance was observed for molecules Ara h 1 (75%), Ara h 3 (75%), and Jug r 2 (70%). The concordance results observed for Ara h 1, Ara h 3, and Jug r 2 may be attributed to the inherent differences in the assay methodologies used by the ISAC and ALEX tests. Specifically, despite the overall concordance for sIgE measured by both tests being relatively high (70% and 75%)), the variability observed in certain measurements may indicate that each test detects distinct epitopes or employs different sensitivity thresholds. For example, while Ara h 3 showed statistical significance in the Mann–Whitney U test (*p* = 0.006), Ara h 1 and Jug r 2 did not present significant differences (*p* > 0.05). Additionally, the analysis using the chi-square test did not show statistically significant differences in outcomes (positive or negative) between the tested assays (*p* = 0.495). This suggests that, in general, both tests provide similar overall classifications, even if individual cases show discrepancies.

Koch et al. also compared the ALEX, ImmunoCAP ISAC, and ImmunoCAP tests in terms of asIgE concordance; however, the comparison was carried out only relative to selected allergen molecules, specifically those with respect to house dust mite components (rDer p 1, 2, 10, and 23). These authors reported test sensitivity to be 85.6%, 86.4%, and 90.8%, respectively, with no significant differences between the three tests. The authors demonstrated high correlations of the asIgE levels relative to Der p 1, Der p 2, and Der p 10 molecules (0.828, 0.807, and 0.969, respectively) in the ALEX and ImmunoCAP ISAC tests [11]. Our present study showed comparable, high correlations of asIgE determinations relative to the above molecules of house dust mites (92.5%, 100%, and 90%, respectively).

A Czech research team (Bojcukova et al.), which compared the ALEX and ImmunoCAP ISAC tests, focused on six selected molecules (Bet v 1, Der p 1, Der p 2, Fel d 1, Phl p 1, and Phl p 5). They reported a Spearman’s rank correlation coefficient ranging from 0.91 (for Fel d 1) to 0.95 (for Bet v 1), which suggests a high concordance of asIgE determinations with the ImmunoCAP ISAC and ALEX tests. There were no significant differences between the two tests in asIgEs relative to these evaluated molecules [13]. In our study, test concordance with respect to asIgEs and relative to the molecules evaluated by Bojcukova et al. was also high and ranged from 87.5% (for Phl p 1) to 100% (for Der p 2).

Klug et al. [12] aimed to demonstrate the prevalence of asIgEs to PR-10 family molecules with the use of ImmunoCAP ISAC and ALEX tests. These authors demonstrated asIgE to Bet v 1 as the most prevalent ones, with the proportion of concordantly positive results at 93.3% (in our study, it was equal to 95%). Out of the PR-10 family molecules assessed by Klug et al., the Gly m 4 molecule demonstrated the lowest concordance (62.5%), and Aln g 1 demnostrated the highest (100%) positive asIgE concordance. In our present study, Gly m 4 asIgE concordance was higher (nearly 90%), and Aln g 1 asIgE concordance was lower (90%) than in the study cited above. Austrian authors reported the noticeably low concordance (at the level of 68%) of Mal d 1 asIgE between the ALEX and ImmunoCAP ISAC tests, whereas the relevant concordance achieved by our research team was 97.5%. The asIgEs relative to Api g 1 also showed a discrepancy (92.5% in our study and slightly over 70% in the study by Klug et al.). Our study on a group of Polish patients also demonstrated higher correlations between asIgEs and molecules Ara h 8 (90%) and Cor a 1.0401 (nearly 90%), obtained via ALEX and ImmunoCAP ISAC tests, than those in the study cited above (slightly over 80% for either molecule).

Heffler et al. assessed the prevalence of asIgE relative to 20 allergen molecules with the ALEX and ImmunoCAP ISAC tests. The highest and the lowest correlation between the ALEX and ImmunoCAP ISAC test was shown for molecules Hev b 8 and Jug r 2, respectively [15]. These findings are also consistent with those of our study, which was conducted in a group of Polish children hospitalized due to food allergy and/or anaphylaxis; the concordance of asIgE levels obtained with the evaluated multiplex tests was nearly 100% in terms of molecule Hev b 8 and 70% in terms of molecule Jug r 2. Moreover, like in the study by Heffler et al., this was the lowest of all assessed asIgE values. These data show that both research teams obtained a high concordance of asIgE determinations with the ALEX and ImmunoCAP ISAC tests.

At the 2020 EAACI congress in London, a Portuguese research team reported a comparison of therapeutic decisions carried out by physicians based on ALEX and ImmunoCAP ISAC test results [10]. A study in patients with symptoms of allergy relative to inhaled allergens (*n* = 19) showed concordant test results in 12 cases (63%). In seven patients, clinically significant sensitization was detected solely via the ImmunoCAP ISAC test; however, it pertained to molecules (Sal k 1, Pla a 2, Pla a 3, Lep d 2, and Can f 5) absent in the then-evaluated version of the ALEX test. Conversely, in eight patients, allergen-inducing inhalation allergy (Alnus, Amaranth, and Glycophagus extracts) was identified solely via the ALEX test. The sensitization profiles of nine patients with food allergy were also assessed. In three of those patients—with a history of allergy to walnuts, sesame seeds, and shrimp—both tests verified sensitization to Jug r 2, Ses i 1, and Pen m 1, respectively. Despite the large number of molecules assessed in both multiplex tests, the specific inducing food could not be identified in three patients with suspected food allergies. In these cases, neither of the evaluated tests proved helpful. In contrast to our findings, the authors of that study emphasized the concordance of asIgE to Jug r 2 in both tests. Those authors also reported false positive results with respect to molecular food allergens (e.g., Act d 1, Ana o 3, Gal d 5, Gad m 1) obtained in both tests, though more commonly in the ALEX test. Due to the observed discrepancies, the authors of the above study stressed the necessity of carefully interpreting asIgE determinations obtained with multiplex tests, with the aim of choosing the most appropriate therapeutic recommendations. They highlighted that oral food challenge tests still remain a gold standard in confirming or excluding food allergies [16].

Analyses of the results obtained in our study demonstrated that positive asIgE determinations obtained in the ALEX test correspond to positive determinations in the ImmunoCAP ISAC test. However, it is worth noting that the absolute asIgE values obtained with either multiplex test are not equivalent. Based on Figure 2, it can be observed that the mean concentration values for the ISAC and ALEX tests differ significantly, which suggests that we should not directly compare the results obtained from these two tests in different units on a 1:1 basis. One of the reasons behind this may be the fact that there are differences in the scaling of values. One of the reasons behind this may be the fact that ALEX is a quantitative test (asIgE levels are expressed in international units: kU_A_/L), whereas ImmunoCAP ISAC is a semi-quantitative test (asIgE levels are expressed in units determined by the manufacturer: ISU). Another reason may be that either test is characterized by a different upper level of quantification; therefore, the mean asIgE values obtained in the ImmunoCAP ISAC test proved to be different from those obtained in the ALEX test (4.1 ISU and 2.0 kU_A_/L, respectively). The discrepant asIgE values obtained with the two tests may also be due to the differences in the way the tests are conducted, such as the use of different allergen matrices, reagent concentrations, and serum sample volumes [15]. In addition, routine ALEX testing is associated with the use of a CCD inhibitor, whereas the procedure for running the ImmunoCAP ISAC test does not include this reagent. The use of a CCD inhibitor in the procedure may eliminate the effect of the amplification of asIgE on allergens that contain carbohydrate determinants in people with anti-CCD antibodies [17,18]. The determinations of asIgEs may be also affected by the use of recombinant molecules. The process of recombinant production may introduce differences in structures (e.g., loss of epitopes) and properties in comparison to natural molecules. The resulting lower number of binding sites for asIgEs may lead to a limited recognition of the given molecule by asIgEs [17]. Out of the 79 molecules evaluated in our study, 19 were derived from a natural source (both in the ALEX and ImmunoCAP ISAC test), whereas 44 molecules were recombinants (in both tests). Nine molecules were natural in the ALEX text and recombinant in the ImmunoCAP ISAC test, whereas in the case of five molecules, the situation was reversed. Two of the evaluated molecules (i.e., Api m 1 and Pru p 3) are a natural–recombinant mix in the ALEX test, whereas both of these molecules are recombinants in the ImmunoCAP ISAC test. In our study, the recommendations of caution in comparing asIgE values seem to apply only to these few determinations. There are studies that introduced positive asIgEs to rAra h 8, rBet v 1, and rPhl p 1 molecules (these were false positive results at 9%, 13%, and 10.5%, respectively) with the ImmunoCAP system, with negative asIgE results relative to these molecules with the ImmunoCAP ISAC system from the same manufacturer [19]. That study drew people’s attention to the matrix onto which the allergens were applied (three-dimensional matrix versus a glass plate), which may be a reason behind the discrepant results for allergen extracts and molecules [19,20,21,22]. Current serological tests play an important role in allergy diagnostics; however, their results—whether for allergen extracts or molecules—should be interpreted with caution [18].

In terms of the number of allergens evaluated via the two diagnostic tests (282 in ALEX and 112 in ImmunoCAP ISAC), each patient in our study had 170 more asIgE determinations conducted with the ALEX test than with the ImmunoCAP ISAC test (this includes both allergen molecules and extracts). This offers a considerable amount of additional information on the patient’s immune status; however, this additional information was not included in the present study. Such analysis may be the topic of another research project.

Previous studies indicate the comparable sensitivity of the ALEX test and that of other multiplex tests, which means that the test is perfectly suitable for use in clinical practice. Our study, conducted in a group of 40 Polish children hospitalized due to food allergy, demonstrated a high correlation between the asIgE results obtained via the ALEX and ImmunoCAP ISAC tests. Like those of authors from other countries, our findings showed that the ALEX test is comparable to the ImmunoCAP ISAC test, with a definite additional advantage in the form of the high number of asIgE determinations obtainable during a single procedure, which provides a plethora of information on the patient’s molecular profile. Another advantage of the ALEX test is the use of an anti-CCD asIgE inhibitor as part of the standard procedure, which minimizes the effect of CCDs on test results of the allergens that naturally contain carbohydrate determinants.

Due to a growing number of commercially available multiplex tests and occasional upgrades of their already existing versions, other research teams are likely to compare the usefulness of those new tests with that of other molecular diagnostic tools in the future. Studies conducted in various populations, including Polish pediatric patients, will provide valuable data on the usefulness of these assessment tools in Central Europe.

### Limitations of This Study

As we present our findings, we are aware that our study is not free of limitations. First, the sample size was relatively small, and the study population only included children with food allergy symptoms. Thus, it cannot be excluded that an analysis of asIgE determinations obtained from a greater number of patients might produce different results. Other factors that may affect the results presented here are the expansion of study assessments to include asIgEs obtained from patients with other clinical manifestations of allergy and the inclusion of adults in the study population. We realize that our study was retrospective in nature, and the presented results were obtained with an earlier version of the ALEX test, which has now been replaced by the ALEX2 test. Moreover, the discrepancies observed in the asIgE values obtained with the ALEX and ImmunoCAP ISAC tests may have been associated with the fact that some of the test molecules were recombinants in one test and natural molecules in the other. Additionally, 30 asIgE results for the ISAC test were above 100 ISU, which may have influenced the range of the obtained results. Furthermore, our study did not include an analysis of clinical utility concerning individual allergens, which is an important area for future investigation. This analysis will be a focal point in our upcoming research.

## 4. Materials and Methods

The study population comprised 40 children with symptoms of food allergy who were hospitalized at the Institute of Tuberculosis and Lung Disease, Department of Allergy and Pulmonology, in Rabka-Zdrój, Poland. There were a total of 28 boys (70%) and 12 girls (30%). The mean age was 8.3 years, with the youngest patient being 2 years old and the oldest being 17 years old. In the study group, there were children, 20 of whom had experienced anaphylaxis in the past. The children also had conditions such as bronchial asthma, atopic dermatitis, oral allergy syndrome (OAS), and allergic rhinitis. Based on the available data, the most common food allergies were relative to cow’s milk proteins, eggs, nuts (including peanuts, hazelnuts, and walnuts), soy, and fruits such as apples and peaches. The children also suffered from respiratory allergies, including those relative to house dust mites; grass (mainly meadow timothy); tree pollen (including birch); and mugwort, animal dander (from cats and dogs), molds, and latex.

Each patient underwent serum asIgE testing with two multiplex tests: ALEX (Macro Array Diagnostics GmbH, Vienna, Austria) and ImmunoCAP ISAC (Thermo Fisher Scientific Inc., Uppsala, Sweden). In total, the collected data comprised 15,760 asIgE determinations, including 4480 values obtained via the ImmunoCAP ISAC test and 11,280 values obtained with the ALEX test. Ultimately, we analyzed 6320 determinations of asIgEs relative to 79 allergen molecules included in both tests. Statistical analyses of data were conducted with the use of SPSS software version 19.0 (IBM Corporation, Somers, NY, USA), and the level of statistical significance was set at *p* = 0.05.

ALEX is a solid-phase immunoassay. Allergen extracts or molecular allergens, which are coupled to nano-particles, are deposited in a systematic fashion onto a solid phase forming a macroscopic array. First, the particle-bound allergens react with a specific IgE that is present in the patient’s sample. During incubation, the asIgEs present in the patient’s serum bind to these allergens. Subsequently, nonspecific, unbound asIgEs are rinsed away. Afterwards, enzyme-labeled anti-human IgE antibodies are added, and they form complexes with the asIgEs bound to allergen molecules. The next steps involve washing and the addition of a substrate. The anti-IgE-bound enzyme converts the substrate into a colored, insoluble product. Finally, a blocking reagent is added to halt enzyme–substrate reactions. The obtained image is analyzed with an ImageXplorer. The whole procedure lasts approximately 3 h and 30 min. The ALEX test yields semi-quantitative determinations of the total IgE (tIgE) levels between 1 kU_A_/L and 2500 kU_A_/L and quantitative determinations of serum asIgE levels between 0.1 kU_A_/L and 50 kU_A_/L. According to the normal ranges provided by the manufacturer, an asIgE level of 0.3 kU_A_/L is considered a positive test result. The quantity of serum required to perform the ALEX test equals 100 µL [7].

The ImmunoCAP ISAC test makes use of allergen components bound to a solid phase. During 120 min of incubation, the asIgEs present in the patient’s serum bind to their corresponding allergen molecules. Subsequently, the plates are washed with solution A and then with demineralized water. Another incubation period, which lasts one hour, takes place after drying the plates and adding the asIgE-detecting solution. The next step involves wiping off the asIgE-detecting solution with a dry paper towel or washing it off with distilled water. At this stage, solution A is added again, and 10 min later, it is washed out. Once the plates are dry, the formation of asIgE–allergen molecule complexes is demonstrated by adding dye-labeled anti-human IgE antibodies. The result is visualized by a scanner. The ImmunoCAP ISAC test manufacturer’s specifications state that asIgE levels must be greater than or equal to 0.3 ISU for the test result to be considered positive. The time required to perform this test is 5 h. The ImmunoCAP ISAC test is conducted with the use of 40 µL serum samples [4,5].

In our study, the results of <0.3 (kU_A_/L and ISU—for ALEX and ImmunoCAP, respectively) were considered negative.

All procedures and methods employed in this study were conducted in strict adherence to the relevant guidelines and regulations, ensuring full compliance with ethical standards and research protocols. This study was approved by the Ethics Committee at the Institute of Tuberculosis and Lung Diseases in Warsaw (approval No. KB-41/2019).

## 5. Conclusions

Our comparison of asIgE values obtained with the ALEX and ImmunoCAP ISAC tests revealed a high concordance of the results, reaching over 90% for a great majority of evaluated molecules. The results of our study support the reliability of the ALEX test in diagnosing sensitization and its effectiveness in assessing asIgEs as part of allergy diagnostics. Nonetheless, there were significant differences between the two tests in the levels of asIgEs relative to individual molecules; this indicates the need for caution in making direct comparisons between asIgE levels obtained with these tests.

## Figures and Tables

**Table 1:** Antigen-specific IgE ImmunoCAP ISAC and ALEX test results, with the use of 79 allergen molecules available in both tests, obtained from 40 Polish children hospitalized due to food allergy/anaphylaxis.

			Test	
			ImmunoCAP ISAC	ALEX
Test results	Negative	Number (*n*)	2493	2515
		Proportion (%)	78.9%	79.6%
	Positive	Number (*n*)	667	645
		Proportion (%)	21.1%	20.4%
Chi-square test of independence			*χ*^2^ = 0.47; *p* = 0.495	

**Table 2:** Antigen-specific IgE determination concordance between the ImmunoCAP ISAC and ALEX tests, with the use of 79 allergen molecules available in both tests, obtained from 40 Polish children hospitalized due to food allergy/anaphylaxis.

	asIgE Results (*n*)	Proportion (%)
ALEX (−) ImmunoCAP ISAC (+)	130	4.1%
ALEX (+) ImmunoCAP ISAC (−)	108	3.4%
Concordant negative (−) results	2385	75.5%
Concordant positive (+) results	537	17.0%

**Table 3:** Concordance between the ImmunoCAP ISAC and ALEX test in the detection of asIgEs relative to 79 allergen molecules available in both tests, obtained from 40 Polish children hospitalized due to food allergy/anaphylaxis.

Concordant asIgE Test Results	Compared Allergen Molecules
100.0%	Ani s 1, Ani s 3, Bla g 5, Can f 1, Der f 2, Der p 2, Fag e 2, Hev b 5, Pla a 1
97.5%	Act d 5, Ara h 9, Bla g 1, Can f 2, Cla h 8, Gal d 3, Hev b 1, Hev b 3, Hev b 8, Mal d 1, Mus m 1, Par j 2, Phl p 5, Phl p 7
95.0%	Alt a 1, Amb a 1, Api m 1, Ara h 2, Asp f 3, Ber e 1, Bet v 1, Bet v 2, Bos d 6, Der f 1, Equ c 1, Fel d 1, Gal d 1, Gal d 5, Gly m 5, Gly m 6, Pen m 1, Phl p 2
92.5%	Act d 2, Api g 1, Ara h 6, Art v 1, Asp f 6, Bos d 8, Can f 3, Che a 1, Der p 1, Fel d 4, Phl p 6, Pol d 5,
90.0%	Act d 1, Aln g 1, Ara h 8, Der p 10, Gal d 2, Ves v 5
87.5%	Cor a 1.0401, Cor a 9, Fel d 2, Gly m 4, Ole e 1, Pla l 1, Phl p 1, Pru p 3, Ses i 1
85.0%	Art v 3, Bos d 4, Bos d 5, Cor a 8, Jug r 1
82.5%	Bla g 2, Cup a 1, Phl p 12
75.0%	Ara h 1, Ara h 3
70.0%	Jug r 2

## Data Availability

All data utilized in this study are held by the corresponding author, E.M. Upon request, these data can be made available for other research purposes.

## Appendix A

**Table A1:** Analysis of Compliance for the 79 Analyzed Common Molecules in ALEX and ISAC Tests.

		ALEX+ ISAC+	ALEX+ ISAC-	ALEX- ISAC+	ALEX- ISAC-
Act d 1	Frequency	6	3	1	30
	% of Row	15.0%	7.5%	2.5%	75.0%
	% of Column	1.1%	2.8%	0.8%	1.3%
Act d 2	Frequency	3	0	3	34
	% of Row	7.5%	0.0%	7.5%	85.0%
	% of Column	0.6%	0.0%	2.3%	1.4%
Act d 5	Frequency	0	0	1	39
	% of Row	0.0%	0.0%	2.5%	97.5%
	% of Column	0.0%	0.0%	0.8%	1.6%
Aln g 1	Frequency	16	4	0	20
	% of Row	40.0%	10.0%	0.0%	50.0%
	% of Column	3.0%	3.7%	0.0%	0.8%
Alt a 1	Frequency	5	1	1	33
	% of Row	12.5%	2.5%	2.5%	82.5%
	% of Column	0.9%	0.9%	0.8%	1.4%
Amb a 1	Frequency	0	2	0	38
	% of Row	0.0%	5.0%	0.0%	95.0%
	% of Column	0.0%	1.9%	0.0%	1.6%
Ani s 1	Frequency	0	0	0	40
	% of Row	0.0%	0.0%	0.0%	100.0%
	% of Column	0.0%	0.0%	0.0%	1.7%
Ani s 3	Frequency	2	0	0	38
	% of Row	5.0%	0.0%	0.0%	95.0%
	% of Column	0.4%	0.0%	0.0%	1.6%
Api g 1	Frequency	7	0	3	30
	% of Row	17.5%	0.0%	7.5%	75.0%
	% of Column	1.3%	0.0%	2.3%	1.3%
Api m 1	Frequency	2	0	2	36
	% of Row	5.0%	0.0%	5.0%	90.0%
	% of Column	0.4%	0.0%	1.5%	1.5%
Ara h 1	Frequency	7	9	1	23
	% of Row	17.5%	22.5%	2.5%	57.5%
	% of Column	1.3%	8.3%	0.8%	1.0%
Ara h 2	Frequency	12	1	1	26
	% of Row	30.0%	2.5%	2.5%	65.0%
	% of Column	2.2%	0.9%	0.8%	1.1%
Ara h 3	Frequency	7	9	1	23
	% of Row	17.5%	22.5%	2.5%	57.5%
	% of Column	1.3%	8.3%	0.8%	1.0%
Ara h 6	Frequency	7	0	3	30
	% of Row	17.5%	0.0%	7.5%	75.0%
	% of Column	1.3%	0.0%	2.3%	1.3%
Ara h 8	Frequency	10	0	4	26
	% of Row	25.0%	0.0%	10.0%	65.0%
	% of Column	1.9%	0.0%	3.1%	1.1%
Ara h 9	Frequency	10	0	1	29
	% of Row	25.0%	0.0%	2.5%	72.5%
	% of Column	1.9%	0.0%	0.8%	1.2%
Art v 1	Frequency	9	2	1	28
	% of Row	22.5%	5.0%	2.5%	70.0%
	% of Column	1.7%	1.9%	0.8%	1.2%
Art v 3	Frequency	5	0	6	29
	% of Row	12.5%	0.0%	15.0%	72.5%
	% of Column	0.9%	0.0%	4.6%	1.2%
Asp f 3	Frequency	0	0	2	38
	% of Row	0.0%	0.0%	5.0%	95.0%
	% of Column	0.0%	0.0%	1.5%	1.6%
Asp f 6	Frequency	0	1	2	37
	% of Row	0.0%	2.5%	5.0%	92.5%
	% of Column	0.0%	0.9%	1.5%	1.6%
Ber e 1	Frequency	1	2	0	37
	% of Row	2.5%	5.0%	0.0%	92.5%
	% of Column	0.2%	1.9%	0.0%	1.6%
Bet v 1	Frequency	22	2	0	16
	% of Row	55.0%	5.0%	0.0%	40.0%
	% of Column	4.1%	1.9%	0.0%	0.7%
Bet v 2	Frequency	12	2	0	26
	% of Row	30.0%	5.0%	0.0%	65.0%
	% of Column	2.2%	1.9%	0.0%	1.1%
Bla g 1	Frequency	1	1	0	38
	% of Row	2.5%	2.5%	0.0%	95.0%
	% of Column	0.2%	0.9%	0.0%	1.6%
Bla g 2	Frequency	0	1	6	33
	% of Row	0.0%	2.5%	15.0%	82.5%
	% of Column	0.0%	0.9%	4.6%	1.4%
Bla g 5	Frequency	0	0	0	40
	% of Row	0.0%	0.0%	0.0%	100.0%
	% of Column	0.0%	0.0%	0.0%	1.7%
Bos d 4	Frequency	9	2	4	25
	% of Row	22.5%	5.0%	10.0%	62.5%
	% of Column	1.7%	1.9%	3.1%	1.0%
Bos d 5	Frequency	10	5	1	24
	% of Row	25.0%	12.5%	2.5%	60.0%
	% of Column	1.9%	4.6%	0.8%	1.0%
Bos d 6	Frequency	10	2	0	28
	% of Row	25.0%	5.0%	0.0%	70.0%
	% of Column	1.9%	1.9%	0.0%	1.2%
Bos d 8	Frequency	13	3	0	24
	% of Row	32.5%	7.5%	0.0%	60.0%
	% of Column	2.4%	2.8%	0.0%	1.0%
Can f 1	Frequency	14	0	0	26
	% of Row	35.0%	0.0%	0.0%	65.0%
	% of Column	2.6%	0.0%	0.0%	1.1%
Can f 2	Frequency	2	0	1	37
	% of Row	5.0%	0.0%	2.5%	92.5%
	% of Column	0.4%	0.0%	0.8%	1.6%
Can f 3	Frequency	5	2	1	32
	% of Row	12.5%	5.0%	2.5%	80.0%
	% of Column	0.9%	1.9%	0.8%	1.3%
Che a 1	Frequency	1	0	3	36
	% of Row	2.5%	0.0%	7.5%	90.0%
	% of Column	0.2%	0.0%	2.3%	1.5%
Cla h 8	Frequency	0	0	1	39
	% of Row	0.0%	0.0%	2.5%	97.5%
	% of Column	0.0%	0.0%	0.8%	1.6%
Cor a 1.0401	Frequency	12	0	5	23
	% of Row	30.0%	0.0%	12.5%	57.5%
	% of Column	2.2%	0.0%	3.8%	1.0%
Cor a 8	Frequency	5	0	6	29
	% of Row	12.5%	0.0%	15.0%	72.5%
	% of Column	0.9%	0.0%	4.6%	1.2%
Cor a 9	Frequency	14	4	1	21
	% of Row	35.0%	10.0%	2.5%	52.5%
	% of Column	2.6%	3.7%	0.8%	0.9%
Cup a 1	Frequency	7	3	4	26
	% of Row	17.5%	7.5%	10.0%	65.0%
	% of Column	1.3%	2.8%	3.1%	1.1%
Der f 1	Frequency	14	0	2	24
	% of Row	35.0%	0.0%	5.0%	60.0%
	% of Column	2.6%	0.0%	1.5%	1.0%
Der f 2	Frequency	17	0	0	23
	% of Row	42.5%	0.0%	0.0%	57.5%
	% of Column	3.2%	0.0%	0.0%	1.0%
Der p 1	Frequency	13	2	1	24
	% of Row	32.5%	5.0%	2.5%	60.0%
	% of Column	2.4%	1.9%	0.8%	1.0%
Der p 10	Frequency	1	1	3	35
	% of Row	2.5%	2.5%	7.5%	87.5%
	% of Column	0.2%	0.9%	2.3%	1.5%
Der p 2	Frequency	17	0	0	23
	% of Row	42.5%	0.0%	0.0%	57.5%
	% of Column	3.2%	0.0%	0.0%	1.0%
Equ c 1	Frequency	2	0	2	36
	% of Row	5.0%	0.0%	5.0%	90.0%
	% of Column	0.4%	0.0%	1.5%	1.5%
Fag e 2	Frequency	0	0	0	40
	% of Row	0.0%	0.0%	0.0%	100.0%
	% of Column	0.0%	0.0%	0.0%	1.7%
Fel d 1	Frequency	13	1	1	25
	% of Row	32.5%	2.5%	2.5%	62.5%
	% of Column	2.4%	0.9%	0.8%	1.0%
Fel d 2	Frequency	6	3	2	29
	% of Row	15.0%	7.5%	5.0%	72.5%
	% of Column	1.1%	2.8%	1.5%	1.2%
Fel d 4	Frequency	2	2	1	35
	% of Row	5.0%	5.0%	2.5%	87.5%
	% of Column	0.4%	1.9%	0.8%	1.5%
Gal d 1	Frequency	15	2	0	23
	% of Row	37.5%	5.0%	0.0%	57.5%
	% of Column	2.8%	1.9%	0.0%	1.0%
Gal d 2	Frequency	14	3	1	22
	% of Row	35.0%	7.5%	2.5%	55.0%
	% of Column	2.6%	2.8%	0.8%	0.9%
Gal d 3	Frequency	8	0	1	31
	% of Row	20.0%	0.0%	2.5%	77.5%
	% of Column	1.5%	0.0%	0.8%	1.3%
Gal d 5	Frequency	5	2	0	33
	% of Row	12.5%	5.0%	0.0%	82.5%
	% of Column	0.9%	1.9%	0.0%	1.4%
Gly m 4	Frequency	9	1	4	26
	% of Row	22.5%	2.5%	10.0%	65.0%
	% of Column	1.7%	0.9%	3.1%	1.1%
Gly m 5	Frequency	3	0	2	35
	% of Row	7.5%	0.0%	5.0%	87.5%
	% of Column	0.6%	0.0%	1.5%	1.5%
Gly m 6	Frequency	14	1	1	24
	% of Row	35.0%	2.5%	2.5%	60.0%
	% of Column	2.6%	0.9%	0.8%	1.0%
Hev b 1	Frequency	0	0	1	39
	% of Row	0.0%	0.0%	2.5%	97.5%
	% of Column	0.0%	0.0%	0.8%	1.6%
Hev b 3	Frequency	0	0	1	39
	% of Row	0.0%	0.0%	2.5%	97.5%
	% of Column	0.0%	0.0%	0.8%	1.6%
Hev b 5	Frequency	0	0	0	40
	% of Row	0.0%	0.0%	0.0%	100.0%
	% of Column	0.0%	0.0%	0.0%	1.7%
Hev b 8	Frequency	12	1	0	27
	% of Row	30.0%	2.5%	0.0%	67.5%
	% of Column	2.2%	0.9%	0.0%	1.1%
Jug r 1	Frequency	7	2	4	27
	% of Row	17.5%	5.0%	10.0%	67.5%
	% of Column	1.3%	1.9%	3.1%	1.1%
Jug r 2	Frequency	8	4	8	20
	% of Row	20.0%	10.0%	20.0%	50.0%
	% of Column	1.5%	3.7%	6.2%	0.8%
Mal d 1	Frequency	16	0	1	23
	% of Row	40.0%	0.0%	2.5%	57.5%
	% of Column	3.0%	0.0%	0.8%	1.0%
Mus m 1	Frequency	8	0	1	31
	% of Row	20.0%	0.0%	2.5%	77.5%
	% of Column	1.5%	0.0%	0.8%	1.3%
Ole e 1	Frequency	2	4	1	33
	% of Row	5.0%	10.0%	2.5%	82.5%
	% of Column	0.4%	3.7%	0.8%	1.4%
Par j 2	Frequency	2	0	1	37
	% of Row	5.0%	0.0%	2.5%	92.5%
	% of Column	0.4%	0.0%	0.8%	1.6%
Pen m 1	Frequency	1	0	2	37
	% of Row	2.5%	0.0%	5.0%	92.5%
	% of Column	0.2%	0.0%	1.5%	1.6%
Phl p 1	Frequency	18	0	5	17
	% of Row	45.0%	0.0%	12.5%	42.5%
	% of Column	3.4%	0.0%	3.8%	0.7%
Phl p 12	Frequency	5	7	0	28
	% of Row	12.5%	17.5%	0.0%	70.0%
	% of Column	0.9%	6.5%	0.0%	1.2%
Phl p 2	Frequency	9	1	1	29
	% of Row	22.5%	2.5%	2.5%	72.5%
	% of Column	1.7%	0.9%	0.8%	1.2%
Phl p 5	Frequency	15	1	0	24
	% of Row	37.5%	2.5%	0.0%	60.0%
	% of Column	2.8%	0.9%	0.0%	1.0%
Phl p 6	Frequency	8	1	2	29
	% of Row	20.0%	2.5%	5.0%	72.5%
	% of Column	1.5%	0.9%	1.5%	1.2%
Phl p 7	Frequency	1	1	0	38
	% of Row	2.5%	2.5%	0.0%	95.0%
	% of Column	0.2%	0.9%	0.0%	1.6%
Pla a 1	Frequency	0	0	0	40
	% of Row	0.0%	0.0%	0.0%	100.0%
	% of Column	0.0%	0.0%	0.0%	1.7%
Pla l 1	Frequency	3	1	4	32
	% of Row	7.5%	2.5%	10.0%	80.0%
	% of Column	0.6%	0.9%	3.1%	1.3%
Pol d 5	Frequency	0	0	3	37
	% of Row	0.0%	0.0%	7.5%	92.5%
	% of Column	0.0%	0.0%	2.3%	1.6%
Pru p 3	Frequency	10	3	2	25
	% of Row	25.0%	7.5%	5.0%	62.5%
	% of Column	1.9%	2.8%	1.5%	1.0%
Ses i 1	Frequency	13	3	2	22
	% of Row	32.5%	7.5%	5.0%	55.0%
	% of Column	2.4%	2.8%	1.5%	0.9%
Ves v 5	Frequency	0	0	4	36
	% of Row	0.0%	0.0%	10.0%	90.0%
	% of Column	0.0%	0.0%	3.1%	1.5%
